# School Engagement in Relation to Body Mass Index and School Achievement in a High-School Age Sample

**DOI:** 10.1155/2018/3729318

**Published:** 2018-10-01

**Authors:** Kristin E. Finn, Myles S. Faith, Young S. Seo

**Affiliations:** ^1^School of Education and Human Services, Canisius College, 2001 Main Street, Buffalo, NY 14208, USA; ^2^Department of Counseling, School, and Educational Psychology, University at Buffalo–State University of New York, 420 Baldy Hall, Buffalo, NY 14260-1000, USA

## Abstract

**Purpose:**

Research has documented an inverse relationship between body mass index (BMI) and school achievement but has failed to empirically explain it. We tested whether this association among adolescents can be explained in part by student engagement.

**Methods:**

A self-report survey about health and school behaviors was completed by 196 high school students; BMI and achievement data were obtained from school records. Three forms of engagement were assessed: behavioral, presenteeism, and affective. Associations of engagement with BMI and achievement were examined, and mediation analyses were conducted.

**Results:**

The simple relationship between BMI and achievement was confirmed and demonstrated that BMI was negatively related to academic achievement. Higher BMI was also significantly correlated with lower classroom participation. Mediation tests showed the significant relationship between BMI and achievement was reduced after accounting for behavioral engagement but not affective engagement.

**Conclusions:**

These novel findings shed light on why heavier students often experience lower academic achievement. Intervention studies targeting barriers to classroom engagement among overweight and obese youth are needed so that their academic potential is not compromised.

## 1. Introduction

Childhood obesity is a major public health problem in the US and around the world. A well-established literature suggested childhood obesity is associated with numerous medical complications and comorbidities [1], psychosocial complications [2], poorer body image [3], and reduced health-related quality of life [4]. In addition to these health complications, emerging interest has focused on the link between obesity and academic achievement. A slowly growing but consistent body of evidence demonstrates that obesity is negatively related to academic performance. A review by Taras and Potts-Datema [5] suggests poorer school performance for children who are overweight or obese for all 10 studies in their review. Indicators of poor performance included lower math and reading scores, IQ scores, grade point average (GPA), educational persistence, absenteeism, grade retention, and placement in special education and remedial classes. Since their review, more recent investigations also consistently demonstrate worse academic outcomes for students with obesity [6, 7]. As educational attainment predicts long-term health outcomes and reduced longevity [8], poorer academic achievement is a concerning comorbidity of childhood obesity.

Despite the prior findings, there is a lack of understanding about the mechanisms underlying the relationship between obesity and achievement [5, 9]. To date, few if any empirical studies have tested the multiple pathways by which obesity affects achievement. A comprehensive review by Caird et al. [10] reported that the association between obesity and educational attainment weakens after controlling for confounding variables such as socioeconomic status. Truong and Sturm [7] conjecture that medical conditions, stigma, and depression may serve as pathways through which obesity affects achievement. Although each factor is plausible, they argue that none of them are sufficient explanations and that most of the association between obesity and education is likely due to other pathways. New data are needed to formally test underlying pathways and mechanisms.

In the present study, we propose that the relationship between obesity and achievement may be mediated, at least in part, by student engagement, a central component of achievement. In the field of education research, “student engagement” is widely regarded as essential for school success. Engagement research and theory has shown tremendous growth over the past two decades [11], with abundant evidence that engagement is a strong predictor of student achievement, motivation, learning, and retention [12, 13]. Engagement is a complex construct and has been conceptualized more fully in the past decade to include affective, behavioral, and cognitive dimensions [14, 15]. In a seminal model of student disengagement, Finn [16] outlined the process of dropping out of school as beginning with a lack of behavioral engagement in school activities (attendance, homework completion, and classroom participation) which then leads to poor school performance and subsequently to a lack of emotional engagement in school (valuing school outcomes and sense of belonging in school). School success (and failure) is the result of a cycle of engagement experiences. This rich literature offers a novel framework for studying the link between childhood obesity and academic performance.

Childhood obesity may in fact have a significant impact on engagement and subsequent academic success. In school settings, students with obesity encounter lower expectations for school success [17, 18]. A recent qualitative review of adolescents in the UK summarized the negative experience of obesity as severe, unrelenting, abusive, and isolating [19]. There is extensive evidence for biases against youth who are obese in schools [20], including from teachers [20] and principals [21] in addition to peers [22]. This may result in disengagement and “presenteeism,” which in the workplace is defined as being present at work, but less productive or not fully functioning due to a health problem [23]. In one study of manufacturing employees, moderately or extremely obese workers experienced greater health-related loss in productivity than those with lower BMIs [24]. Studies of costs associated with lost productivity show that workers who are obese miss more work days due to illness, injury, or disability [25]. Similarly, students who are obese compared to healthy weight may be “showing up for work” (i.e., school attendance) but with diminished engagement on academic tasks. Empirical tests of the relationship between obesity and engagement are beginning to emerge. For example, in a recent study using the National Survey of Children's Health, adolescents with obesity were significantly more likely to be absent from school and less engaged [26].

While it is theoretically reasonable, to our knowledge, no studies have directly assessed the pathway between obesity, engagement, and achievement. Further, there is a lack of consistency in the way that educational experiences such as engagement and achievement have been operationalized. For instance, engagement has been measured as failing grades and unexplained absences [27] as well as parent's reports of their child caring about doing well in school and completing required work [26]. In this study, engagement is conceptualized as proschool attitudes and behaviors that contribute to success in school; it is operationalized as having both external (behavioral) and internal (affective) factors. The purpose of this investigation was to test whether the relationship between child BMI and academic achievement is mediated by behavioral and affective indicators of school engagement.

## 2. Method

### 2.1. Participants

A sample of 196 students from a large suburban high school in the northeast were included in the present study. Once the study gained approval from the Internal Review Board from Canisius College, participants completed surveys during three separate periods of a health class during the school day. Most (95%) of the participants were in grade 9 (average age 14.65). The sample was evenly split between males (47%) and females (53%). About 70% of the respondents reported race as white, 15% as African American, 6% Hispanic, and 9% other.

### 2.2. Measures

Three forms of school engagement were assessed: behavioral, presenteeism, and affective. These measures represent engagement ranging from more specific, external classroom behaviors and actions (behavioral) to internal attitudes and values about school and education (affective).

#### 2.2.1. Behavioral Engagement/Participation

Behavioral engagement in school was assessed with 6 items that measured the extent to which students participate in the classroom. Example items include “I pay attention in class,” “I participate in class discussions,” and “I come to class on time.” The items were adapted from Finn's school participation questionnaire (see [28]). Each item used a 4-point Likert scale ranging from “strongly disagree” to “strongly agree.” The internal reliability coefficient for the scale was 0.82 in this sample.

#### 2.2.2. Presenteeism

Presenteeism was assessed with a 7-item scale adapted from the World Health Organization's Health and Work Performance Questionnaire [29]. The scale assessed the extent to which students reported limited performance at school during the past month (e.g., not working carefully, lower work quality, and performance limited by health problems). This form of engagement was generally external, but less specific and classroom-related than the participation engagement measure. Each item was assessed on a 4-point scale that ranged from “none of the time” to “most of the time.” The internal reliability estimate for the scale was 0.78 in this sample.

#### 2.2.3. Affective Engagement/Identification

Affective engagement was assessed with the 10-item identification with school questionnaire [30]. The measure indicates the degree to which students value both school and school-related outcomes and have a sense of belongingness in their school. Identification with school represents an internal form of engagement. Each item was assessed on a 4-point scale that ranged from “strongly disagree” to “strongly agree.” Example items included “I feel proud of being part of my school,” “I am treated with as much respect as other students in my class,” and “School is one of the most important things in my life.” The scale has good psychometric properties with an internal reliability coefficient of 0.79 in this sample.

#### 2.2.4. GPA

Academic achievement was provided by school records indicating the student's current overall grades (GPAs) for their current year in school. Scores ranged from 61.58 to 99.56, with average GPA of 86.46 (standard deviation 9.03).

#### 2.2.5. Body Mass Index

BMI (kg/m^2^) for each student was obtained from school records. BMI was derived from children's weights and heights which were collected by New York State-certified physical education teachers during fitness testing which was part of the normal fitness unit curriculum at the beginning of the school year. A balance scale was used to measure weight, and a wall tape measure was used to record height. Body mass index for students, as reported by the school, ranged from 14.7 to 48.7 with an average BMI of 22.84 (standard deviation 5.5).

### 2.3. Data Analysis

Because obesity and school achievement are often associated with demographic characteristics, mediation analyses controlled for gender and race [7, 32]. The purpose of the present study was to examine whether engagement in school can be used to explain the link between obesity and achievement among adolescents. Specifically, regression analysis was used to test whether this relationship was mediated by three different indicators of school engagement.

Mediation analyses were conducted using SPSS PROCESS v.3 macro [33] to examine whether the relationship between student BMI and academic achievement was mediated through student participation, presenteeism, and identification with school. Specifically, a parallel multiple mediator model was employed by putting all three potential mediators simultaneously, thus allowing us to examine multiple mechanisms at the same time and to compare the magnitude of the indirect effects of student BMI through them. Compared with the traditional mediation approach suggested by Baron and Kenny [32], the total effect of the predictor on the outcome (here student BMI on GPA) may be obtained by summing the direct effect and specific indirect effects.

**Figure 1:**
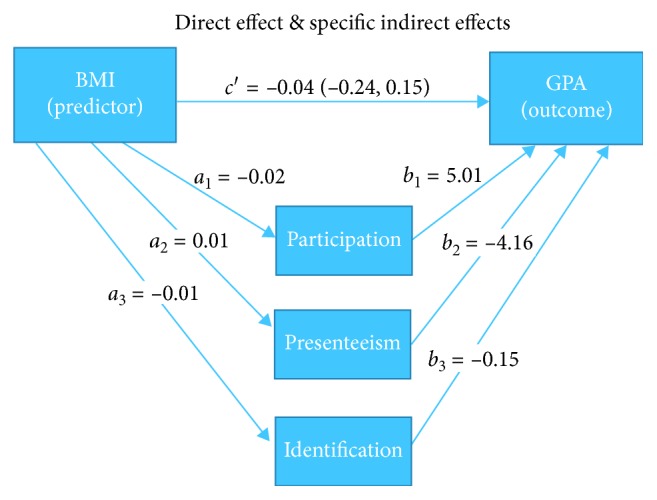
Parallel multiple mediation model tested through a set of paths controlling for gender and race. Note: direct effect (*c*′) = −0.04 (−0.24, 0.15); specific indirect effect of participation (*a*_1_*b*_1_) = −0.09 (−0.18, −0.01); specific indirect effect of presenteeism (*a*_2_*b*_2_) = −0.05 (−0.14, 0.04); specific indirect effect of identification (*a*_3_*b*_3_) = 0.00 (−0.02, 0.03); total effect (*c*)=*c*′+(*a*_1_*b*_1_+*a*_2_*b*_2_+*a*_3_*b*_3_).

In Hayes' approach [33], both the direct and the indirect effects are obtained by estimating the coefficients from the three paths simultaneously as follows (Figure 1). First, student GPAs are regressed on student BMI while holding all three mediators, student gender, and race (white vs. non-white) constant (direct effect (*c*′)). Second, student BMI predicts student participation at school (*a*_1_), which in turn predicts student GPA (*b*_1_) (specific indirect effect 1 (*a*_1_*b*_1_)). Third, student BMI predicts student presenteeism at school (*a*_2_), which in turn predicts student GPA (*b*_2_) (specific indirect effect 2 (*a*_2_*b*_2_)). Fourth, student BMI predicts student identification with school (*a*_3_), which in turn predicts student GPA (*b*_3_) (specific indirect effect 3 (*a*_3_*b*_3_)). Eventually, the total effect (not shown here) can be computed by adding direct effect (*c*′) to the sum of specific indirect effects (*a*_1_*b*_1_+*a*_2_*b*_2_+*a*_3_*b*_3_). In order to check the robustness of the indirect effects, the bias-corrected bootstrap confidence intervals were computed which were derived by taking a random sample with 100,000 replacements.

## 3. Results

### 3.1. Descriptives

Descriptive statistics and correlation coefficients for the sample are given in Table 1. Gender was not significantly correlated with any of the other variables. Race was significantly correlated with both BMI and GPA; lower BMI and higher GPA were more typically associated with white students. Greater classroom participation and less presenteeism were also more likely among white students. Race was unrelated to level of school identification. Average student BMI for the sample was 22.84. The average BMI breakdown by gender and race was 22.7 for males, 22.9 for females, 22.1 for white, and 24.3 for nonwhite.

As expected, the inverse correlation between BMI and GPA was statistically significant (*r*=−0.17, *p*=0.019) for the entire sample. The correlations further reveal important relationships for the three engagement measures. All three forms of engagement were interrelated with correlations ranging from 0.42 to 0.66. All forms of engagement measures were also significantly related to GPA, demonstrating the importance of engagement for academic success. Greater achievement was found for students with higher levels of classroom participation (0.53) and school identification (0.28) and lower levels of presenteeism (−0.51). In terms of BMI, the only engagement predictor to be significant was classroom participation (i.e., behavioral engagement) such that higher levels of BMI were associated with lower classroom participation (*r*=−0.21).

### 3.2. Partitions of Direct Effect and Indirect Effects

The direct effect of BMI, *c*′ = −0.04, is the estimated differences in GPAs between students having the same school engagement (participation, presenteeism, and identification), gender, and race but differ by one unit in their BMI. As shown in Table 2, student GPAs did not differ as a function of student BMI when controlling for participation, presenteeism, identification, student gender, and race (*c*′ = −0.04, *t*(186) = −0.44, *p*=0.66, CI (−0.24, 0.15)). Thus, the association between BMI and GPA is nonsignificant after accounting for engagement mediators and control variables.

The indirect effects show the relationships between BMI and GPA as mediated by the effect of participation, presenteeism, and identification. The results of mediation analyses in Table 2 indicated that student BMI was inversely related to student participation at school (*a*_1_=−0.02, SE=0.01, *p* < 0.05), which in turn was associated with a decline in student GPAs (*b*_1_=5.01, SE=1.49, *p*=0.001). The indirect effect of student BMI on GPAs as modeled through student behavioral engagement (participation at school) was estimated as *a*_1_*b*_1_ = −0.02(5.01) = −0.09. Students with a one unit higher BMI exhibited lower behavioral engagement (by 0.09 units), which in turn was associated with a decrease in their GPAs (bias-corrected bootstrap CI (−0.18, −0.01)). Thus, the relationship between BMI and GPA was significantly mediated by participation.

Indirect effects involving the other two engagement predictors showed that BMI was not significantly related to either presenteeism (*a*_2_=0.01, SE=0.001, *p* > 0.05) or identification (*a*_3_=−0.01, SE=0.01, *p* > 0.05). The relationship between presenteeism and GPA was significant (*b*_2_=−4.16, SE=1.14, *p* < 0.001) such that lower achievement was found for students who reported lower work quality at school. There was no significant relationship between identification with school and GPA (*b*_3_=−0.15, SE=1.40, *p* > 0.05). Despite the relationship between presenteeism and GPA, there was no evidence of an indirect effect of presenteeism on the link between BMI and GPA. Similarly, no evidence was found to suggest that identification with school was a mediator of this relationship. Thus, the association between BMI and achievement was not influenced by student presenteeism at school (*a*_2_*b*_2_ = −0.05, bias-corrected bootstrap CI (−0.14, 0.04)) and/or identification with school (*a*_3_*b*_3_ = −0.00, bias-corrected bootstrap CI (−0.02, 0.03)).

## 4. Discussion

As discussed previously, there is ample evidence to show that being overweight or obese is related to poorer educational outcomes. In particular, research consistently finds an inverse relationship between BMI and academic achievement. What is unclear, however, is the causal explanation for this relationship. Given the widespread evidence that school engagement is necessary for academic success, we hypothesized that students who are overweight or obese may be less engaged in school which, in turn, explains why these students have worse academic outcomes. This hypothesis was tested with several forms of engagement among students.

Overall, our results confirmed that BMI was related to GPA and that student engagement was a significant mediator of this relationship. These are important findings since research so far has failed to clarify this relationship. Despite that all three forms of engagement were associated with achievement, our mediation hypotheses were only supported for the most behavioral form of engagement (i.e., classroom participation). Presenteeism in school and the affective form of engagement did not help to explain the effect of BMI on GPA. This suggests that specific, observable student behaviors in school such as participating in class discussions, handing in assignments, and putting forth effort are more important than internal attitudes about the value of school and feeling a sense of belonging. This is consistent with engagement research that shows that while attitudes about school are important predictors of school achievement, attitudes and perceptions are not sufficient alone and are most effective when coupled with academic behaviors [16]. Shernoff [34] argues that participation in schooling is essential for educational success and goes so far as to say “take away participation, and there can be no benefit of schooling” (p.10). Since all of our measures of engagement were intercorrelated and related to achievement, future research should examine the overlapping and unique effects of different types of engagement. A serial multiple mediator model could highlight potentially complex relationships among the mediators. It may be useful to establish how one engagement mediator affects another. For instance, does participation lead to identification or vice versa?

There are several possible reasons for why students with obesity may be less engaged in school. For instance, it is possible that weight bias may prevent or discourage students who are obese from being fully engaged in school. Obese compared to healthy weight children are more likely to be bullied in school. Such stigma and bullying among adolescents who are obese may result in diminished social, psychological, and physical health [35]. Students who feel ashamed and embarrassed may be reluctant to participate in class discussion or may avoid classes such as physical education. Students who are teased, bullied, or rejected may withdraw from others or avoid coming to school altogether. One study reported that the odds of skipping school because of weight-based teasing increased 5% per teasing incident for students who experience weight-based victimization [36]. In a study of 84 children enrolling in a family-based obesity treatment study, child-reported teasing/social rejection was associated with poorer child general academic ability as reported by teachers [37].

In addition, poor health may also contribute to lack of engagement. Daniels [38] review of the impact of obesity on school outcomes points to several health-related reasons for poor performance. Obesity-related problems such as poor nutrition, sedentary lifestyle, asthma, orthopedic problems, and sleep apnea may affect a student's ability to pay attention and stay focused in class, may impair cognitive functioning, contribute to mood disruptions, and increase tardiness and absenteeism. Students who have obesity-related health problems may miss school days because they do not feel well or have medical appointments. Sleep difficulties may make it difficult to arrive at school on time or pay attention in class. One study found that children referred by physicians to a special clinic for severely obese students missed a median of one day from school in the preceding month [39]. Additionally, Pan et al. [40] found that adolescents with obesity missed 37% more sick days due to health than adolescents with normal weight. A recent review of health and achievement shows that students with poor physical fitness and who do not maintain a healthy weight are at risk for poor school performance [9]. While not assessed in this study, it may be worthwhile to explore weight bias and health-related quality of life as impediments to school engagement in future investigations.

Limitations of this study include the inability to make causal connections with correlational data. For example, any causal effect of BMI on school achievement may be due in part to weight bias exposure. Mediation analyses shed light on the probable connection between obesity, engagement, and achievement; however, experimental or longitudinal designs would provide more definitive explanations. Also, the use of self-report data may result in biased estimates of behavioral engagement and work habits should be corroborated with teacher observations when possible. On the other hand, judgments of effort and concentration may be best assessed through self-report, and predictor and outcome variables were both obtained through school records. It should also be mentioned that students completed the survey during health classes which may have influenced some responses. Although most engagement items pertained to general school attitudes and behaviors, a small number of items on the presenteeism scale addressed limitations due to health issues which may have been more threatening for students who were overweight.

Our findings reveal an opportunity for novel intervention studies targeting barriers to school engagement among youth who are overweight or obese, such that academic potential is realized and not compromised. We are unaware of any such research, which could occur at a schoolwide level or target individual youth. There have been “universal” antibullying campaigns that have some successes in improving school climate [41]. School-based campaigns combating weight bias (e.g., discrimination among teachers and/or students) have not been studied to our knowledge but could be investigated. Alternatively, interventions might target specific youth who are weight teased, emotionally distressed from these experiences, and consequently disengaged in the classroom. Coping skills interventions for weight teasing have received only preliminary investigation [42] and could be studied in schools, especially if academic performance is compromised. Greater collaborations among education and obesity researchers could help cultivate new intervention research.

In summary, this study offers novel insight into an important and unresolved issue. To our knowledge, this is the first study to successfully test and identify engagement as an explanation for why achievement is threatened for students with obesity. Both status (e.g., race/ethnicity) and academic (e.g., grade retention) factors have been identified that place students at risk for educational failure and dropping out. Resilient students are those individuals who can overcome these barriers and succeed despite their level of risk. A large, influential study found impressive differences between resilient and nonresilient students on several measures of school engagement [12]. Thus, school engagement may be regarded as a protective factor in terms of educational risk [28]. Students who attend class, pay attention to the teacher, persist on difficult tasks, and complete assignments increase their likelihood of school success. Perhaps it is time to better recognize obesity as a risk factor for poorer achievement, and target students who are obese and less engaged in school and start to develop interventions that encourage engagement behaviors that foster daily learning.

## Figures and Tables

**Table 1:** Correlations among all variables.

	Gender	Race	BMI	GPA	Participation	Presenteeism	Identification
Gender	1.00	0.12	0.02	0.06	−0.05	0.03	−0.08
Race		1.00	0.18^*∗*^	−0.29^*∗∗*^	−0.24^*∗∗*^	0.17^*∗*^	−0.02
BMI			1.00	−0.17^*∗*^	−0.21^*∗∗*^	0.13	−0.06
GPA				1.00	0.53^*∗∗*^	−0.51^*∗∗*^	0.28^*∗∗*^
Participation					1.00	−0.66^*∗∗*^	0.57^*∗∗*^
Presenteeism						1.00	−0.42^*∗∗*^
Identification							1.00
*Mean*			22.84	86.46	3.15	2.00	3.03
*Standard deviation*			5.49	9.03	0.54	0.62	0.47

*Note*. Gender: 0 = male and 1 = female; race: 0 = white and 1 = nonwhite. ^*∗*^*p* < 0.05; ^*∗∗*^*p* < 0.01.

**Table 2:** Regression analyses predicting GPA from BMI, participation, presenteeism, and identification.

Antecedent	Consequent															
	*M*_1_(participation)				*M*_2_(presenteeism)				*M*_3_(identification)				*Y* (GPA)			
		*b*	SE	*p*		*b*	SE	*p*		*b*	SE	*p*		*b*	SE	*p*
*X* (BMI)	*a*_1_	−0.02	0.01	0.012	*a*_2_	0.01	0.01	0.142	*a*_3_	−0.01	0.01	0.405	*c*′	−0.04	0.10	0.661
*M*_1_(participation)		—	—	—		—	—	—		—	—	—	*b*_1_	5.01	1.49	0.001
*M*_2_(presenteeism)		—	—	—		—	—	—		—	—	—	*b*_2_	−4.16	1.14	<0.001
*M*_3_(identification)		—	—	—		—	—	—		—	—	—	*b*_3_	−0.15	1.40	0.916
*Covariate: female*		−0.04	0.08	0.598		0.02	0.09	0.862		−0.08	0.07	0.263		1.79	1.07	0.096
*Covariate: nonwhite*		−0.25	0.08	0.004		0.20	0.10	0.043		−0.00	0.08	0.973		−3.47	1.21	<0.01
Constant	*i*_*M*_1__	3.64	0.16	<0.001	*i*_*M*_2__	1.66	0.19	0.001	*i*_*M*_3__	3.19	0.15	<0.001	*i*_*Y*_	80.56	7.01	0.001
	*R* ^2^ = 0.092				*R* ^2^ = 0.040				*R* ^2^ = 0.011				*R* ^2^ = 0.362			
	*F* (3,189) = 6.40, *p* < 0.001				*F* (3,189) = 2.62, *p*=0.052				*F* (3,189) = 0.69, *p*=0.557				*F* (6,186) = 17.58, *p* < 0.001			

*Note*. Control variables included student gender and race.

## Data Availability

The data used to support the findings of this study were provided through confidential school records under an agreement with the author and so cannot be made freely available. Access to these data will be considered by the author upon request, with permission of the cooperating school district.
